# Difficult reversal of dabigatran with idarucizumab in a multiple-trauma patient: A question of dose?

**DOI:** 10.1016/j.tcr.2021.100422

**Published:** 2021-02-10

**Authors:** Mathias Ströhle, Christopher Rugg, Stefan Schmid, Dietmar Fries, Elgar Oswald

**Affiliations:** Department of Anaesthesiology and Critical Care Medicine, Medical University of Innsbruck, Anichstrasse 35, 6020 Innsbruck, Austria

**Keywords:** Antidote, Idarucizumab, Dabigatran, Multiple trauma, Bleeding

## Abstract

Dabigatran is an oral anticoagulant directly acting as thrombin inhibitor. The monoclonal antibody idarucizumab was developed to reverse its anticoagulatory effects after application of a standardized dose. Following administration, dabigatran plasma level rebounds have been reported but its consequences are not fully understood. We report a case of a multiple-trauma patient under dabigatran treatment suffering from secondary bleeding relapse after initially successful reversal with idarucizumab. Stabilisation of the patient's coagulopathy and subsequent bleeding was not achieved until application of an additional dose of idarucizumab. We conclude that patients treated with dabigatran and presenting with active bleeding require close attention to its reversal with standard doses of idarucizumab. Screening for thrombin time was shown beneficial in early detection of dabigatran rebound in this case.

## Introduction

Dabigatran, a direct thrombin inhibitor, is primarily eliminated in the urine with a terminal half-life of 12–17 h in healthy patients. Its anticoagulatory effects can be rapidly reversed with idarucizumab which binds both free and thrombin-bound dabigatran (two bolus injections of 2.5 g reduce dabigatran plasma concentrations >99% [1]) [2]. The terminal half-life of idarucizumab is 10.3 h. The REVERSE-AD trial reported a dabigatran plasma level rebound in 23% of all cases resulting in 2% bleeding relapse following idarucizumab treatment [2]. Other case reports have described dabigatran rebound 7 to 61 h after initial idarucizumab administration requiring repeated dosage [2,3]. Being a hydrophilic molecule, dabigatran can easily move between intra- and extravascular compartments and it is thought that a variable intravascular concentration may account for rebound bleeding accompanied by fluctuating coagulation parameters. Therefore, a single dose of idarucizumab might not be sufficient to completely neutralise dabigatran's effects [4].

## Case

We report our experience with a poly-trauma patient whose medical management was likely compromised by severe coagulopathy secondary to dabigatran. This 83-year-old Caucasian male (83 kg, 180 cm) fell from a ladder and suffered multiple injuries resulting in an injury severity score of 34. He had been prescribed dabigatran 110 mg bid since 2011 for atrial fibrillation, recurrent pulmonary embolism (2003 and 2011) and transient ischemic attacks (2018). The last dose of dabigatran was 9 h prior to trauma. Blood creatinine was 1.5 mg/dl. Other medications included bisoprolol 1.25 mg qd, lisinopril 10 mg qd, simvastatin 40 mg qd, pantoprazole 40 mg qd and allopurinol 300 mg qd. Further comorbidities were hypertension, hyperlipidaemia, gout and an inguinal hernia. No allergies were reported, and the patient did not have a history of easy bleeding/bruising prior to dabigatran treatment. INR prior to trauma was between 1.4 and 2.3 (0.8–1.3).

The patient was initially treated in the ER of a county hospital. An arterial and central venous line were placed, the patient was intubated and ventilated. The contrast-enhanced whole-body CT indicated a subarachnoid 9 mm bleeding, bilateral haematopneumothoraces with lung contusions, left sided rib fractures II-XII, bilateral clavicular fractures, I-III lumbar fracture, minor spleen lesion and a psoas hematoma. Both thoracic cavities were drained. The patient was transfused with 1500 ml balanced crystalloids (ELO-MEL®, Fresenius, Germany), 500 ml succinylated gelatine (Gelofusin®, BBraun, Germany) and 2 units of packed red blood cells (PRBCs) and required intravenous administration of norepinephrine (>0.25 μg kg-1 min-1) to maintain stable hemodynamic parameters (Table 1). Blood work indicated a prolonged aPTT (activated partial thromboplastin time) and elevated INR (international normalised ratio; Table 1). Medical treatment included administration of fibrinogen concentrate 2 g (Haemocomplettan®, CSL Behring, Germany), tranexamic acid 1 g and idarucizumab 5 g, as recommended. The bleeding situation improved markedly. Due to injury severity the patient was transferred to our Level I trauma centre. On arrival, blood work showed only slightly prolonged aPTT and TT (thrombin time). Coagulation tests used, were Pathromtin SL assay® for aPTT, Thromborel S assay® for PT, BC Thrombin assay® for TT and Multifibren U (Clauss) assay® for fibrinogen, all Siemens, Marburg, Germany. Lowered fibrinogen was substituted with another dose of 2 g. Fluid resuscitation included administration of 500 ml of crystalloids, 500 ml of succinylated gelatine, and one unit of PRBC.

**Table 1:** Timeline of treatment, blood samples and patient condition.

	Time of accident	Arrival minor trauma Center		Arrival major trauma Center	Arrival ICU department							
	Day 1							Day 2				
	16:00	17:30	18:00	21:00	21:25	22:30	23:19	00:16	00:51	01:19	05:23	13:53
Blood values												
Thrombin time (*sec*, 15–21)					28		89			29	26	20
aPTT (sec, 26–37)		60			49		185			57	49	46
INR (PT)		1.7			1.6		2.0			1.6	1.5	1.4
Fibrinogen (mg/ dl, 210–400)					75		103			208	220	242
Plt (g l^−1^, 150–380)		148			94		79			84	73	65
Hgb (g dl^−1^, 13.0–17.7)		12.7			11.2		8.1			8.1	9.2	9.7
FXIII (%, 70–140)					61		52			57	71	55
Lactate (mmol l^−1^, 0.63–2.44)		1.77			2.44		2.33			1.11	3.55	1.88
Dabigatran (ng ml^−1^)												<20

ROTEM												
INTEM CT (s, 100–240)					192		172			169	172	
CFT (s, 30–110)					223		174			138	133	
MCF (mm, 50–72)					50		53			54	53	
LI30 (%, 94–100)					100		100			100	100	
LI60 (%, 85–100)					100		100			99	98	
A10 (mm, 44–66)					35		39			41	43	
EXTEM CT (s, 38–79)					83		61			57	50	
CFT (s, 34–159)					178		159			131	117	
MCF (mm, 50–72)					52		54			56	58	
LI30 (%, 94–100)					100		100			100	100	
LI60 (%, 85–100)					100		100			100	99	
A10 (mm, 43–65)					39		41			44	47	
FIBTEM MCF (mm, 9–25)					4		9			16	16	
A10 (mm, 7–23)					4		7			13	14	

Treatment												
Crystalloids (ml)			1500		500							
Colloids (ml)			500		500		500	500		500	500	
PRBC			2			1		3				
PC								1				
Fibrinogen Concentrate (g)			2			2		4				
4F-PCC (IE)								1200				
Factor XIII Concentrate								1250				
Tranexamic Acid (g)			1					1				
Idarucizumab (g)			5						2.5			
Norepinephrine (µg kg^−1^ min^−1^)	0.25	0.3	0.4	0.4	0.4	0.4	0.3	0.3	0.25	0.1	0.1	0.1
Chest drain effusion (ml)		~1000					150	300	650			

aPTT = activated Partial Thromboplastin Time, INR = International Normalised Ratio; Plt = Platelets, Hgb = Haemoglobin, ROTEM = rotational thromboelastometry; CT = Clotting Time; CFT = Clot Forming Time; MCF = Maximum Clot Firmness; LI30 = Lysis Index 30 min; LI60 = Lysis Index 60 min; A10 = Amplitude 10 min; PRBC = packed red blood cell; PC = platelet concentrate; 4F-PCC = 4-factor prothrombin complex concentrate.

About 5 h after recommended dosage of idarucizumab, blood work indicated a relapse in prolongation of aPTT and TT. Blood loss through the chest drains increased from zero up to 1100 ml in 90 min (Table 1). Intravenous norepinephrine and fluid requirements increased. One unit of platelet concentrate (PC), three units of PRBCs, fibrinogen concentrate 4 g, 4F-PCC 1200 IU (Prothromplex Total®, Baxter, Vienna), Factor XIII concentrate 1250 IU (Fibrogammin®, CSL Behring, Germany), and tranexamic acid 1 g were administered primarily suspecting traumatic bleeding. Despite this intensive medical management following the European trauma guidelines, stabilisation was not achieved [5,6]. Finally, another half-dose of idarucizumab (2.5 g) was given, to treat the possibility of incomplete reversal of dabigatran-induced coagulopathy. Hereafter, the patient's condition stabilised promptly. With decreased catecholamine requirement and bleeding cessation we resigned from completing to a second full-dose of 5 g [7]. Laboratory work-up showed almost normal aPTT and TT values, further improving over the next 12 h (Table 1). Dabigatran plasma levels 12 h after 7.5 g of idarucizumab were beneath detectability, indicating sufficient long-term reversal. The patient was referred back to the county hospital after 17 days with subsequent transfer to a rehabilitation facility two weeks later.

## Discussion

In this case, dabigatran serum levels were not measured sequentially. The link to ongoing bleeding and abnormal coagulation values is therefor made more difficult. Although plasma level concentrations of dabigatran may not be useful for measuring the antagonistic effects of idarucizumab [3] they can be used to predict possible dabigatran rebound when measured prior to administration [8]. Bleeding-related coagulopathy was treated according to the rotational thrombelastometry results (ROTEMdelta®, Instrumentation Laboratory, Bedford MA, USA). While ROTEM maximal clot firmness stays unaffected, other haemostatic tests relying on thrombin (e.g. aPTT, TT, fibrinogen) are inaccurate in the presence of high dabigatran levels due to thrombin inactivation [4]. Other considerable factors maybe contributing to the coagulopathic state of this patient include dilutional coagulopathy, disseminated intravascular coagulation and trauma-related coagulopathy. They can be excluded by the ROTEM findings or missing triggers. Factor-specific inheritable deficiencies and liver or renal failure are not supported by history or laboratory values. Finally, our suspicion was confirmed by prompt cessation of bleeding as well as hemodynamic stabilisation after another half-dose of idarucizumab.

## Conclusion

We conclude that a dabigatran-related coagulopathy contributed to massive bleeding in a multiple traumatised patient. An increasing number of patients admitted to the ER are treated with directly acting oral thrombin inhibitors. In the absence of feasible plasma level testing, screening for aPTT and TT can be beneficial for identifying patients at risk but can also support early detection of non-responders to antidote therapy. Patients treated with dabigatran and presenting with active bleeding require close attention to its reversal with standard doses of idarucizumab.

## CRediT authorship contribution statement

**MS** designed the study, collected and interpreted the data and drafted the article. **CR** and **SS** contributed to data collection and drafted the article. **DF** and **EO** revised the article and contributed to the final article.

## Patient consent

The patient in this manuscript has given written informed consent to publication of his case details.

## Assistance with the article

None.

## Financial support and sponsorship

This research received no specific grant / financial support or sponsorship from any funding agency in the public, commercial or not-for-profit sectors.

## Declaration of competing interest

**MS**, **CR**, **SS** and **EO** none. **DF** has received personal fees for lectures or similar in the past, independent of the presented manuscript. **DF** has received personal fees from 10.13039/100008322CSL Behring GmbH, from LFB, and BBraun and non-financial support from TEM International, outside the submitted work.
